# Effect of genetic ancestry on leukocyte global DNA methylation in cancer patients

**DOI:** 10.1186/s12885-015-1461-0

**Published:** 2015-05-27

**Authors:** Mónica Cappetta, María Berdasco, Jimena Hochmann, Carolina Bonilla, Mónica Sans, Pedro C Hidalgo, Nora Artagaveytia, Rick Kittles, Miguel Martínez, Manel Esteller, Bernardo Bertoni

**Affiliations:** 1 Departamento de Genética, Facultad de Medicina, Universidad de la República, Montevideo, Uruguay; 2 Cancer Epigenetics and Biology Program (PEBC), Bellvitge Biomedical Research Institute (IDIBELL), 08908 L’Hospitalet de LLobregat, Barcelona, Catalonia Spain; 3 School of Social and Community Medicine, University of Bristol, Bristol, UK; 4 Departamento de Antropología Biológica, Facultad de Humanidades y Ciencias de la Educación, Universidad de la República, Montevideo, Uruguay; 5 Centro Universitario de Tacuarembó, Universidad de la República, Tacuarembó, Uruguay; 6 Departamento Básico de Medicina, Facultad de Medicina, Universidad de la República, Montevideo, Uruguay; 7 Department of Surgery and Public Health, University of Arizona, Tucson, USA; 8 Cátedra de Dermatología, Hospital de Clínicas “Manuel Quintela”, Universidad de la República, Montevideo, Uruguay; 9 Department of Physiological Sciences II, School of Medicine, University of Barcelona, Barcelona, Spain; 10 Institucio Catalana de Recerca i Estudis Avançats (ICREA), Barcelona, Catalonia Spain

**Keywords:** Genetic ancestry, DNA methylation, Admixture, Cancer

## Abstract

**Background:**

The study of genetic variants alone is not enough to explain a complex disease like cancer. Alterations in DNA methylation patterns have been associated with different types of tumor. In order to detect markers of susceptibility for the development of cutaneous melanoma and breast cancer in the Uruguayan population, we integrated genetic and epigenetic information of patients and controls.

**Methods:**

We performed two case–control studies that included 49 individuals with sporadic cutaneous melanoma and 73 unaffected controls, and 179 women with sporadic breast cancer and 209 women controls. We determined the level of global leukocyte DNA methylation using relative quantification of 5mdC by HPLC, and we compared methylation levels between cases and controls with nonparametric statistical tests. Since the Uruguayan population is admixed and both melanoma and breast cancer have very high incidences in Uruguay compared to other populations, we examined whether individual ancestry influences global leucocyte DNA methylation status. We carried out a correlation analysis between the percentage of African, European and Native American individual ancestries, determined using 59 ancestry informative markers, and global DNA methylation in all participants.

**Results:**

We detected global DNA hypomethylation in leukocytes of melanoma and breast cancer patients compared with healthy controls (p < 0.001). Additionally, we found a negative correlation between African ancestry and global DNA methylation in cancer patients (p <0.005).

**Conclusions:**

These results support the potential use of global DNA methylation as a biomarker for cancer risk. In addition, our findings suggest that the ancestral genome structure generated by the admixture process influences DNA methylation patterns, and underscore the importance of considering genetic ancestry as a modifying factor in epigenetic association studies in admixed populations such as Latino ones.

**Electronic supplementary material:**

The online version of this article (doi:10.1186/s12885-015-1461-0) contains supplementary material, which is available to authorized users.

## Background

DNA methylation is a critical epigenetic modification of the genome and is involved in regulating many cellular processes including gene expression and genomic stability. Not surprisingly, a growing number of human diseases are associated with alterations in DNA methylation [1]. Deregulation of epigenetic modification in tumor DNA such as hypermethylation of CpG islands at gene promoters and global reduction of 5-methylcytosine (5mC) levels has been observed in almost every cancer type [2, 3]. Although DNA methylation profiles are often tissue- and cell-specific, recent data indicate that epigenetic traits in white blood cells are phenotypic markers of genomic instability and promising candidate risk markers for solid tumors even after adjusting for known cancer risk factors [4, 5].

There is evidence that ethnic groups differ in terms of their patterns of DNA methylation in healthy and tumor tissues [6–10]. Lower global levels of DNA methylation among healthy middle-aged African American women relative to European Americans have been reported [6], and some differences may be already present at birth [11]. Moreover, the rates of incidence of some epigenetically influenced diseases, such as cancer, differ among ethnic groups, due to different environmental exposures, lifestyles and genetic or epigenetic variants.

For instance, breast cancer incidence varies substantially across ethnic groups in the US. In addition, genetic ancestry is associated with breast cancer risk in US Latinas and Mexican women, where higher European ancestry was associated with increased risk and higher Native American ancestry was associated with decreased risk of breast cancer [12, 13]. The overall incidence of cutaneous melanoma has been increasing continuously for the last four decades in European populations and populations of European descent [14]. Similarly to breast cancer, European ethnicity was associated with an increased risk of cutaneous melanoma in Brazil [15], and we have previously detected an excess of European ancestry in Uruguayan melanoma patients (J.H., unpublished data). Very little has been published regarding cutaneous melanoma in other admixed populations.

The Uruguayan population has been described fundamentally as of European origin. However, genetic admixture analysis has shown that it is a tri-hybrid population with genetic contributions from Native Americans and Africans as well [16, 17].

Breast cancer is the most common type of cancer among women in Uruguay. The national incidence rate is 90.7/100,000 women per year (age-adjusted rates) [18]. These rates are the highest in Latin America and resemble those seen in developed Western countries [19]. The age-adjusted national incidence rates for melanoma are 4.5 and 3.5 per 100.000 in men and women respectively, and are clearly on the rise since a previous study conducted in 1996 [20].

Only a few studies have examined the association between ethnicity and DNA methylation in cancer patients, all of them were based on self-reported ethnicity and most investigated DNA methylation changes occurring at tissue level in normal and diseased state [21]. In order to detect susceptibility markers of sporadic cutaneous melanoma and breast cancer in the Uruguayan population, we examined genetic and epigenetic information in melanoma and breast cancer patients and controls. In particular, we assessed global DNA methylation in leukocytes of sporadic cancer patients and its association with individual genetic ancestry.

## Methods

### Study population

We performed two case–control studies: 49 individuals with sporadic cutaneous melanoma and 73 unaffected controls were recruited at Hospital de Clínicas “Dr. Manuel Quintela” (Montevideo), and 179 women with sporadic breast cancer and 209 women controls were enrolled in different health institutions across Uruguay. All individuals participating in the breast cancer study were originally recruited in a previous study described in Bonilla et al. [22].

In both studies all patients and controls were genetically unrelated Uruguayans without family history of skin cancers, and breast or ovarian cancer, respectively. All breast cancer patients had been diagnosed a year or less before inclusion in the study, while melanoma patients were recruited at diagnosis. None of the patients had been subjected to radiotherapy or chemotherapy close to the time of blood draw. Controls were selected from the same hospitals as patients. Breast cancer controls were women over 35 years of age with a normal mammogram. All participants in both case–control studies were required to have a normal hemogram for inclusion in the study.

The procedures followed in both studies were approved by the ethics committee of the Facultad de Medicina of the Universidad de la República, Uruguay. After obtaining written informed consent from all participants of the study, peripheral blood was drawn for DNA extraction and participants answered an interview-based questionnaire to record medical and epidemiological information.

### Global DNA methylation analysis

We measured global DNA methylation levels in leukocytes through relative quantification of 5-methyl 2′-deoxycytidine (5mdC) using liquid chromatography by HPLC as detailed elsewhere [23]. Briefly, DNA was hydrolyzed with nuclease P1 (Sigma-Aldrich) and alkaline phosphatase (Fermentas-Thermo Scientific) to yield 2′-deoxymononucleosides, which were separated by HPLC and detected by ultraviolet (UV) light. A mixture of deoxyadenosine, deoxythymidine, deoxyguanosine, deoxycytidine, 5-methyl-2′-deoxycytidine and deoxyuridine (Sigma-Aldrich) was used as a standard. The percentages of global genomic DNA methylation were calculated by integration of the 5mdC peak area (obtained directly from the HPLC) relative to global cytidine (methylated or not).

A subset of 49 melanoma patients and 60 unaffected controls, and 95 breast cancer patients and 95 unaffected women were analyzed by HPLC in duplicate. The average for each sample was calculated. Duplicated samples showing a difference in 5mdC greater than 3 % or with low HPLC resolution were removed.

### Genotyping and Individual admixture analysis

The ancestry informative markers (AIMs) used in this study were 59 biallelic single nucleotide polymorphisms (SNPs) (Additional file 1: Table S1) selected from the AIMs panel for Hispanic populations described by Fejerman et al. [12] which show a large difference in allele frequency between ancestral populations (>0.5). The AIMs are spaced along the 22 autosomes to assure balanced ancestry information. The AIMs were genotyped by the KASPar SNP Genotyping System (Kbiosciences Ltd, UK). Individual genetic ancestry was analyzed using the Bayesian Markov Chain Monte Carlo algorithm implemented in STRUCTURE 2.3.4 [24]. Given the trihybrid parental contribution (European, African and Native American) to the Uruguayan population the program was run mainly with K = 3, but also with K = 2 due to the African contribution being quite low, as the predefined setting for the number of ancestral populations, with 10,000 iterations for the burn-in period and 50,000 additional iterations to obtain parameter estimates. In all cases the program was instructed to use parental population information. Several options were explored, such as the admixture and linkage models, and independent or correlated allele frequencies, to uncover changes in the clustering pattern. The AIMs data from parental populations used to estimate admixture proportions included 42 Europeans, 37 West Africans, and 30 Native Americans (15 Mayans and 15 Nahuas), which were genotyped on an Affymetrix 100K SNP chip (data kindly provided by Dr. Laura Fejerman, UCSF).

In order to test for association of genes involved in DNA methylation with global DNA methylation, we genotyped SNPs in MTHFR (C677T rs1801131), DNMT3A (rs4665777), DNMT3B (rs406193) and BRCA1 (rs16942, rs1799950, rs176092, rs8176193) in all breast cancer cases and controls with methylation level data.

We analyzed methylation levels around each AIM in populations closely related to the parental populations of the Uruguayan samples to better understand the relationship between ancestry and global DNA methylation. Methylation data was obtained from 96 African Americans (AA), 96 Caucasian Americans (CA) and 96 Han Chinese Americans (HC), used in the Heyn et al. study [7]. A window of 100kb surrounding each AIM was analyzed, the average methylation status was calculated for the whole window and also for promoters, gene bodies and intergenic regions.

### Statistical analysis

Shapiro-Wilks test was used to test for normality of the methylation data. Because methylation data were not normally distributed, we used nonparametric tests in the statistical analyses. We applied the Mann–Whitney-Wilcoxon test to identify differences between affected and control individuals in binary variables or the Kruskall-Wallis test for variables with more than two states. The epidemiologic variables analyzed are shown in Additional file 2: Table S2.

We also used logistic regression to examine the association of methylation data with disease, adjusted by age and ancestry. To evaluate the effect of potential confounders of the association between global DNA methylation and cancer, we examined the association of disease status with age, smoking status, body mass index (BMI) and genetic variants associated to epigenetic processes in breast cancer study. BMI and smoking status are not considered risk factors for melanoma in the literature, so we measured only age and gender as a confounders in our sample.

The association between DNA methylation and individual ancestry was assessed with the Kendall rank correlation test. To visualize the relationship between individual ancestry and DNA methylation we used a classification and regression tree [25]. We performed all statistical analyses using the R environment for statistical computing version 2.15.3 [26].

## Results

We measured global genomic methylation levels in leukocytes through relative quantification of 5mdC in cutaneous melanoma and breast cancer patients and unaffected control individuals. We obtained global DNA methylation data for 42 melanoma patients and 46 controls as well as for 86 breast cancer patients and 92 controls (data available in Additional files 3 and 4).

In both case–control studies performed, we found a significant difference in global leukocyte DNA methylation between individuals with cancer and unaffected controls (p < 0.001; Table 1). The average methylation levels in melanoma and breast cancer patients were lower (2.54 ± 0.37 % and 2.33 ± 0.48 %, respectively) than the average methylation levels in unaffected controls (2.79 ± 0.27 and 2.77 ± 0.77 %, respectively), (Table 1 and Additional file 5: Figure S1). We found evidence of a difference in age between cases and controls (Additional file 2: Table S2). Therefore, we analyzed the correlation between global DNA methylation levels and age in both the melanoma and the breast cancer studies and did not detect a significant association (Table 2). No other associations with confounding factors were uncovered for cancer status or global DNA methylation (Table 1 and Additional file 2: Table S2). We did not detect an association between genetic variants directly or indirectly involved in epigenetic processes such as C677T in *MTHFR*, rs4665777 in *DNMT3A*, rs406193 in *DNMT3B*, rs16942, rs1799950, rs8176092, rs8176193in *BRCA1*, and global DNA methylation levels in the breast cancer study (p > 0.05 for all, Additional file 6: Table S3).

**Table 1:** Average global DNA methylation and ancestral contributions in melanoma and breast cancer cases and controls

	N	Cases	N	Controls	P value
*Global DNA methylation (±SD)*					
Melanoma^a^	42	2.54 ± 0.37 %	46	2.79 ± 0.27 %	9.96e^−4^
Breast cancer^a^	86	2.33 ± 0.48 %	92	2.77 ± 0.77 %	5.96e^−5^
*Global DNA methylation-Gender*					
Melanoma					
Female	22	2.48 ± 0.42 %	29	2.79 ± 0.24 %	0.269^b^
Male	20	2.63 ± 0.30 %	17	2.80 ± 0.32 %	0.198^c^
*Ancestry (*±SD)					
Melanoma	49		73		
European		95.04 ± 6.48 %		93.32 ± 11.39 %	0.184
Native American		3.33 ± 5.81 %		4.94 ± 7.71 %	0.211
African		1.16 ± 3.37 %		1.74 ± 4.84 %	0.120
Breast cancer	179		209		
European		76.89 ± 12.95 %		76.49 ± 14.26 %	0.927
Native American		12.86 ± 10.46 %		13.85 ± 11.37 %	0.229
African		10.25 ± 8.30 %		9.66 ± 7.55 %	0.420

^a^Logistic regression for Melanoma P = 7.10e^−4^ and Breast cancer P = 5.47e^−4^, adjusted by ancestry and age

^b^Comparison between females and males in cases.

^c^Comparison between females and males in controls

**Table 2:** Kendall correlation coefficients for the relationship between leukocyte global DNA methylation and ancestral components and age

	N^a^	Age	N^a^	European	Native American	African
*Melanoma*	86	−0,023				
Cases	41	−0.140	28	0.184	−0.204	−0.132
Controls	45	−0.034	39	−0.125	0.153	0.000
*Breast cancer*	169	−0.028				
Cases	80	−0.008	78	0.124	−0.062	−0.199^b^
Controls	89	0.064	89	−0.024	0.080	−0.074
*Total samples*						
Cases	121	−0.049	106	0.169^b^	−0.127	−0.187^c^
Controls	134	0.072	128	0.017	0.045	−0.098

^a^Individuals with DNA methylation data but without age and/or ancestry data were removed from this analysis

^**b**^p < 0.01

^**c**^p < 0.005

In order to determine the degree of admixture of all subjects, we analyzed a set of 59 SNPs that can identify Native American, African, and European ancestry (Additional file 1: Table S1). In the admixture analysis, we first studied the ancestry distribution among individuals, and there was no significant difference in the ancestral individual contributions between melanoma patients and unaffected controls. The same was found in the breast cancer case–control study (Table 1). However, we detected higher percentages of European ancestral contribution in the melanoma case–control study than in the breast cancer study. The differences between the two case–control studies may be attributable to differences in sampling locations. We performed a logistic regression of global DNA methylation and disease status adjusted by ancestry and age to avoid confounder effects and we found that the association was still significant for melanoma and breast cancer (P = 7.10e-4 and P = 5.47e-4 respectively; Table 1).

To ascertain whether individual ancestry is influencing the global DNA methylation status in leukocytes, we carried out a correlation analysis between the percentage of African, European and Native American individual contributions and global DNA methylation. As shown in Table 2, the Kendall rank correlation revealed a significant inverse association between the African ancestral component and the percentage of global DNA leukocyte methylation in breast cancer patients (τ = −0.199, p < 0.01). We did not observe a significant association between methylation and genetic ancestry in melanoma patients probably due to the reduced numbers of participants without missing data. However, when considering all melanoma and breast cancer patients together, the negative correlation with the African ancestral component became statistically stronger (τ = −0.187 p < 0.005, Table 2 and Additional file 7: Figure S2a). A significant positive correlation with the European component was also found (τ = 0.169 p < 0.01, Table 2 and Additional file 7: Figure S2b). In contrast, we could not detect any correlation between DNA methylation level and genetic ancestry in unaffected controls.

The relationship between the AIMs and methylation status was analyzed in AA, CA and HC populations. The methylation level in intergenic regions shows statistically significant differences between these 3 populations (Table 3). Average DNA methylation in gene bodies and promoter regions in the CA population also exhibits differences with respect to AA and HC. The average DNA methylation levels of CpG sites in a ±100 kb flanking region for each AIM shows that 27 of 48 AIMs are different between at least two populations (Additional file 8: Table S4).

**Table 3:** Mean difference in CpG methylation in a ±100 kb window flanking ancestry informative markers (AIMs) between African-Americans (AA), Caucasian-Americans (CA) and Han Chinese-Americans (HC), according to gene context

Gene Context	Mean β value				P value^*^	
	AA	CA	HC	AA_CA	AA_HC	CA_HC
Gene body	0.520	0.497	0.523	4.85e^−6^	3.56e^−1^	4.20e^−8^
Intergenic region	0.584	0.552	0.594	2.81e^−17^	2.48e^−3^	4.24e^−31^
Promoter	0.304	0.292	0.306	1.92e^−2^	6.72e^−2^	2.77e^−5^

*Wilcoxon Rank Sum test, FDR p-value

**Fig. 1:**
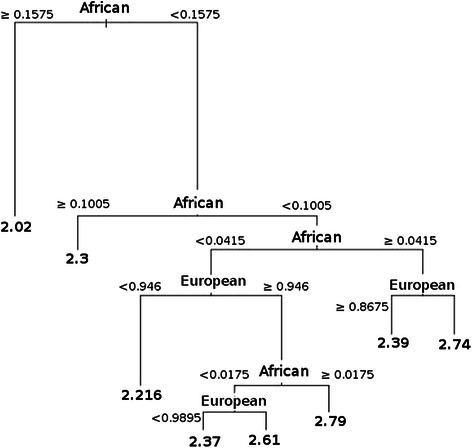
Regression tree analysis of leukocyte global DNA methylation and ancestry in cancer patients. Ancestry is shown as a frequency, and predictive global DNA methylation percentages are shown at the end of the branches.

A clearer image of the relationship between ancestry and global methylation was obtained using a classification and regression tree of disease status on DNA methylation, adjusted by age. The tree shows the predictive values or averages of DNA methylation according to the proportion of African and European ancestry in each patient (Fig. 1). The higher the African genetic individual component in patients, the lower the percentage of DNA leukocyte methylation detected.

## Discussion

There is growing evidence that leukocyte DNA methylation status is associated with cancer [4, 5]. The potential causes of this association include environmental and genetic factors. In the present work, we investigated whether genetic ancestry plays a role on the patterning of global DNA methylation.

We have uncovered evidence of global DNA hypomethylation in leukocytes of cancer patients and of a negative correlation between African genetic individual ancestry and leucocyte methylation among sporadic cancer patients from an admixed population. Previous studies have identified differences in DNA methylation between ethnic groups, regardless of disease status [6–8, 10, 11]. But in all of these studies ethnicity was self-reported. To the best of our knowledge no other studies have examined the relationship between individual genetic ancestry, calculated using AIMs, on global leukocyte DNA methylation.

Genomic DNA hypomethylation is believed to have an important impact on tumor biology through the generation of chromosomal instability, reactivation of transposable elements, and loss of imprinting [27]. Thus, an association between genomic hypomethylation and cancer was expected. There is increasing evidence that leukocytes may be a useful cell model to evaluate epigenetic changes. Epimutations and global DNA hypomethylation, associated with increased cancer risk could be detected in peripheral blood, instead of the affected tissue [5]. This is important since blood samples are much easier to obtain and can be used for large-scale epidemiological studies [28].

We report a significant association of global leukocyte DNA hypomethylation with sporadic cutaneous melanoma. This result is in contrast to a previous study, carried out in melanoma-prone families, which found no significant association between overall or CpG site-specific LINE-1 methylation in peripheral blood and cutaneous melanoma [29]. However, there are two important differences between the studies. First, the methylation levels of one repetitive LINE-1 element do not necessarily represent 5mdC content across the whole genome. And second, the study by Hyland et al. examined the association between DNA methylation and familial cutaneous melanoma, suggesting that in this case the genetic mechanism was more important than the epigenetic effect. On the other hand, we corroborated earlier findings of an association of leucocyte DNA hypomethylation with breast cancer risk [30]. The level of global DNA methylation found in our study is slightly lower than some previous studies reported. However, the overall level of DNA methylation in blood samples varies substantially (2.3 to 6 %) depending on the protocols used, the disease considered, the age range and a number of environmental factors [31–36]. Therefore, the challenge to use global hypomethylation as a clinical marker in cancer will be to define and standardize methodologies for its determination in large numbers of patients.

Growing evidence shows that global DNA methylation, particularly in blood, changes with age, gender, BMI, and lifestyle factors, such as diet and smoking [37, 38]. However, global methylation levels were not associated with any of the epidemiologic variables examined in our study.

At the population level, the incidence rates of some complex diseases like cancer vary between populations from different continents, probably as a result of adaptation to local selective factors but also as a result of genetic ancestry. Ethnic differences in breast cancer and cutaneous melanoma incidence are well documented and in both cases European descendants show higher rates with respect to other geographic populations [18]. Therefore, the trihybrid genetic structure of the Uruguayan population is ideal for studying the effects of different ancestral genetic components on disease. It has been recently reported that slight ethnic differences exist at birth among specific CpGs, and the predominant pattern is of lower levels of CpG methylation among African Americans [11]. Then, the negative correlation between African ancestry and global genomic methylation in leukocytes of cancer patients found in the present study is in agreement with the existing literature. Since we observed no differences between patients and controls with respect to individual genetic ancestry, DNA hypomethylation in patients cannot be attributed to differences in genetic ancestry between groups.

When analyzing CpGs surrounding the AIMs we observed a significant difference between CA and other populations (i.e. AA and HC). It has been demonstrated that methylation levels at individual CpG sites can be strongly associated with both local and distant sequence variation [39, 40]. SNP allele frequencies can differ substantially among populations with different geographic ancestries [41], which suggest that ethnic differences in DNA methylation could be due to differences in population specific alleles or haplotypes that influence CpG and global methylation levels. It seems that even though there is no ancestry difference between patients and controls, the former have exclusive African sequences associated with hypomethylation. Consequently, the individual genetic ancestry may contribute to the inter-individual variation of global DNA methylation among cancer patients.

Fraser et al. [42] suggested that DNA methylation is highly divergent between populations, and that this divergence may be in large part due to a combination of differences in allele frequencies and complex epistasis or gene-environment interactions. Thus, a variant that is present in two populations could affect DNA methylation in only one. Moreover, Heyn et al. [7] identified DNA methylation differences that distinguish three major human ethnic groups (Caucasian-American, African-American and Han Chinese-American) and CpG methylation quantitative trait loci associated with natural human variation, contributing to the diverse phenotypic characteristics of human populations.

There is limited data available on DNA methylation in worldwide populations. Even though the Han Chinese is not strictly a parental population to modern Uruguayans, it is the closest to Native Americans we can get with data available for both the AIMs (HapMap) and CpG methylation status [7]. Since we wanted to explore the relationship between the AIMs and the methylation status of CpGs surrounding the AIMs in the parental populations, we used the Han Chinese as a proxy for Native Americans. The genetic distance between East Asians and Native Americans is smaller than between Africans or Europeans and Native Americans [43]. The statistical differences in DNA methylation levels reported by Heyn et al. [7] and our analysis of their data, between African Americans, Caucasian Americans and Han Chinese, suggest that there may also exist differences between the Uruguayan parental populations. Therefore, the tri-hybrid structure of the admixed Uruguayan population could partially explain the methylation pattern. The African genetic ancestry may play a particular role in the susceptibility to or etiology of these cancers, not necessarily in the same way as in other regions of the world.

These results should be confirmed in large cohort studies, given the relatively small sample size of our case–control groups, as well as in other admixed populations. We had no data on cancer staging or tumor grade, so additional studies are required to clarify if the influence of individual genetic ancestry on DNA methylation levels affects tumor aggressiveness or outcome in patients with different ancestral components.

## Conclusions

In conclusion, our findings suggest that individual genetic ancestry influences global leukocyte DNA methylation level in cancer patients of Uruguay. These findings highlight the importance of taking into account individual genetic ancestry when examining epigenetic data in Latin American populations. Most importantly, the possibility that genetic ancestry could be associated to methylation or demethylation-prone chromosomal regions indicates that the admixture gene mapping technique [44] could be extended to relate ancestral chromosomal segments, epigenetic status and susceptibility to disease. Thus, the prevalence of epigenetic alterations may provide a basis for understanding the unequal cancer burden of disease early onset, aggressiveness, and poor outcomes experienced by individuals of different ethnicities.

### Availability of supporting data

The data sets supporting the results of this article are included within the article and its additional files.

## Additional files

Additional file 1: Table S1. List of ancestry informative markers (AIMs) used to determine individual genetic ancestry.
Additional file 2: Table S2. Epidemiologic characteristics evaluated in cancer patients and unaffected controls with global DNA methylation data.
Additional file 3:
**Raw data of global DNA methylation, genetic ancestry and epidemiological variants evaluated in melanoma case–control study.**

Additional file 4:
**Raw data of global DNA methylation and genetic ancestry of breast cancer case–control study.**

Additional file 5: Figure S1. Comparison of the percentage of global DNA methylation levels in leukocytes of cancer patients and unaffected controls. The breast cancer case–control study is shown in red. The cutaneous melanoma case–control study is shown in blue. The box represents the interquartile range and the line across the box indicates the median value. Statistically significant differences between cancer patients and healthy controls were determined using the Wilcoxon Rank Sum test (* = p < 0.001).
Additional file 6: Table S3. Association between SNPs in genes directly or indirectly involved in epigenetic processes and global DNA methylation in leukocytes of individual of the breast cancer study.
Additional file 7: Figure S2. Correlation between individual genetic ancestry and global DNA methylation in leukocytes of cancer patients. (a) The African ancestry component was negatively correlated with DNA methylation (r = −0.187, p < 0.005). (b) The European ancestry component was positively correlated with DNA methylation (r = 0.169, p < 0.01).
Additional file 8: Table S4. Mean DNA methylation level (β) of CpG sites flanking AIMs in African Americans (AA), Caucasian Americans (CA) and Han Chinese Americans (HC).

## Abbreviations

5mC: 5-methylcytosine
5mdC: 5-methyl 2′-deoxycytidine
HPLC: High-performance liquid chromatography
AIMs: Ancestry informative markers
SNP: Single nucleotide polymorphism
BMI: Body mass index
AA: African Americans
CA: Caucasian Americans
HC: Han Chinese Americans
