# Supplementary material for: Effect of genetic ancestry on leukocyte global DNA methylation in cancer patients
Source: BMC Cancer. 2015 May 27;15:434. doi: 10.1186/s12885-015-1461-0 (PMC4445803; doi:10.1186/s12885-015-1461-0)
Supplement: Additional file 1: Table S1. — List of ancestry informative markers (AIMs) used to determine individual genetic ancestry. [file 12885_2015_1461_MOESM1_ESM.doc]

**ADDITIONAL FILE 1**

| **AIMs** | **Chromosome** | **Alleles** |
| --- | --- | --- |
| rs1934393 | 1 | C/G |
| rs2817611 | 1 | A/G |
| rs3828121 | 1 | C/T |
| rs6684063 | 1 | G/T |
| rs10498255 | 2 | C/T |
| rs1470524 | 2 | C/T |
| rs3860446 | 2 | C/T |
| rs842634 | 2 | C/T |
| rs868179 | 2 | A/G |
| rs1395771 | 3 | A/G |
| rs1984473 | 3 | C/T |
| rs6804094 | 3 | A/T |
| rs9310888 | 3 | A/G |
| rs1398829 | 4 | A/T |
| rs9307613 | 4 | A/T |
| rs10515535 | 5 | A/G |
| rs257748 | 5 | A/T |
| rs10484578 | 6 | A/G |
| rs6569792 | 6 | A/G |
| rs6911727 | 6 | C/T |
| rs9320808 | 6 | A/G |
| rs10214949 | 7 | A/G |
| rs10486576 | 7 | C/T |
| rs4733652 | 8 | C/T |
| rs9325872 | 8 | A/G |
| rs10491654 | 9 | C/T |
| rs4013967 | 9 | C/T |
| rs10508349 | 10 | A/G |
| rs1397618 | 10 | A/T |
| rs10501474 | 11 | C/T |
| rs879780 | 11 | C/T |
| rs948360 | 11 | A/G |
| rs28931575 | 11 | ALU/- |
| rs4034627 | 12 | C/T |
| rs4076700 | 12 | C/T |
| rs4762106 | 12 | A/G |
| rs10492585 | 13 | C/T |
| rs2585901 | 13 | C/T |
| rs10131076 | 14 | A/G |
| rs1451928 | 14 | G/T |
| rs9323178 | 14 | A/G |
| rs10520678 | 15 | C/T |
| rs9302185 | 15 | C/T |
| rs1426654 | 15 | A/G |
| rs1004704 | 16 | A/G |
| rs10500505 | 16 | A/T |
| rs30125 | 16 | A/G |
| rs3138523 | 16 | ALU/- |
| rs4130513 | 16 | C/T |
| rs10491097 | 17 | A/G |
| rs2253624 | 17 | G/T |
| rs1013459 | 18 | A/G |
| rs12953952 | 18 | A/G |
| rs798887 | 19 | A/G |
| rs888861 | 19 | A/G |
| rs2208139 | 20 | C/T |
| rs708915 | 20 | A/T |
| rs2829454 | 21 | A/G |
| rs138022 | 22 | A/G |

**Table S1.** List of ancestry informative markers (AIMs) used to determine individual genetic ancestry.
