# Supplementary material for: Effect of genetic ancestry on leukocyte global DNA methylation in cancer patients
Source: BMC Cancer. 2015 May 27;15:434. doi: 10.1186/s12885-015-1461-0 (PMC4445803; doi:10.1186/s12885-015-1461-0)
Supplement: Additional file 2: Table S2. — Epidemiologic characteristics evaluated in cancer patients and unaffected controls with global DNA methylation data. [file 12885_2015_1461_MOESM2_ESM.doc]

**ADDITIONAL FILE 2**

**Table S2. Epidemiologic characteristics evaluated in cancer patients and unaffected controls with global DNA methylation data.**

|  | **N*** | **Cases** | **N*** | **Controls** | **p value** |
| --- | --- | --- | --- | --- | --- |
| ***Age mean (range)*** |  |  |  |  |  |
| Melanoma  Breast cancer | 41  80 | 57 (24-82)  58 (36-85) | 45  89 | 65 (31-82)  53 (36-72) | 0.011^c^  0.0007^c^ |
| ***Gender (%) ^a^***  Female  Male | 42 | 53.7  46.3 | 46 | 64.4  35.6 | 0.3811^d^ |
| ***BMI (%) ^b^***  <25  25-29,9  >30 | 69 | 36.2  34.8  29.0 | 87 | 39.1  32.2  28.7 | 0.9186^d^ |
| ***Smoking status (%) ^b^***  smoker  non-smoker | 81 | 42.0  58.0 | 89 | 50.5  49.5 | 0.4599^d^ |

* Data on age, BMI and smoking status was not available for all individuals in the study.

^a^ Melanoma case-control study

^b^ BMI and smoking status were only evaluated in the breast cancer study.

^c^ Student test p-value

^d^ Fisher test p-value
