# Supplementary material for: Effect of genetic ancestry on leukocyte global DNA methylation in cancer patients
Source: BMC Cancer. 2015 May 27;15:434. doi: 10.1186/s12885-015-1461-0 (PMC4445803; doi:10.1186/s12885-015-1461-0)
Supplement: Additional file 5: Figure S1. — Comparison of the percentage of global DNA methylation levels in leukocytes of cancer patients and unaffected controls. The breast cancer case–control study is shown in red. The cutaneous melanoma case–control study is shown in blue. The box represents the interquartile range and the line across the box indicates the median value. Statistically significant differences between cancer patients and healthy controls were determined using the Wilcoxon Rank Sum test (* = p < 0.001). [file 12885_2015_1461_MOESM5_ESM.doc]

**ADDITIONAL FILE 5**


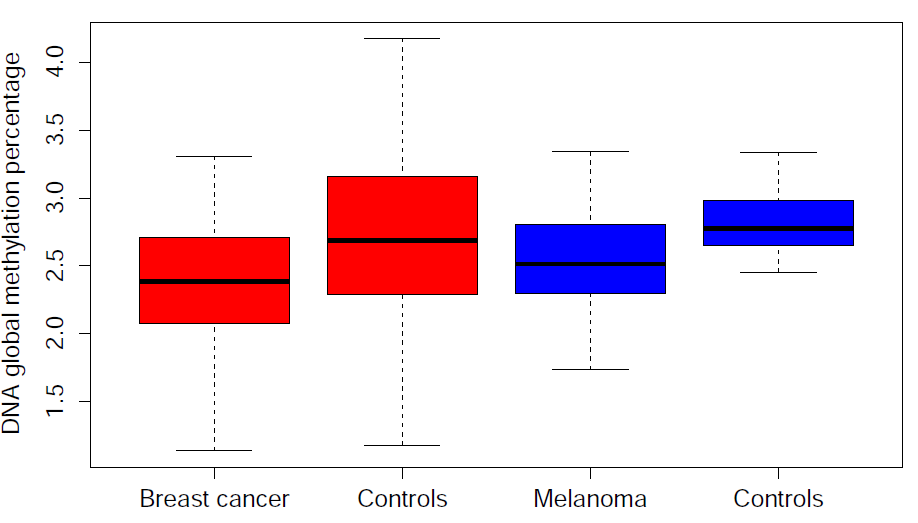


*

*

**Figure S1.** **Comparison of the percentage of global DNA methylation levels in leukocytes of cancer patients and unaffected controls.** The breast cancer case-control study is shown in red. The cutaneous melanoma case-control study is shown in blue. The box represents the interquartile range and the line across the box indicates the median value. Statistically significant differences between cancer patients and healthy controls were determined using the Wilcoxon Rank Sum test (* = p<0.001).
