# Supplementary material for: Effect of genetic ancestry on leukocyte global DNA methylation in cancer patients
Source: BMC Cancer. 2015 May 27;15:434. doi: 10.1186/s12885-015-1461-0 (PMC4445803; doi:10.1186/s12885-015-1461-0)
Supplement: Additional file 6: Table S3. — Association between SNPs in genes directly or indirectly involved in epigenetic processes and global DNA methylation in leukocytes of individual of the breast cancer study. [file 12885_2015_1461_MOESM6_ESM.docx]

**Additional file 6:**

Table S3. **Association between SNPs in genes directly or indirectly involved in epigenetic processes and global DNA methylation in leukocytes of individual of the breast cancer study.**

| **SNPs** | **Chromosome** | **GENE** | **p value** |
| --- | --- | --- | --- |
| rs1801133 | 1 | MTHFR | 0.981 |
| rs4665777 | 2 | DNMT3A | 0.165 |
| rs16942 | 17 | BRCA1 | 0.733 |
| rs1799950 | 17 | BRCA1 | 0.731 |
| rs8176092 | 17 | BRCA1 | 0.672 |
| rs8176193 | 17 | BRCA1 | 0.570 |
| rs406193 | 20 | DNMT3B | 0.447 |
