# Supplementary material for: Effect of genetic ancestry on leukocyte global DNA methylation in cancer patients
Source: BMC Cancer. 2015 May 27;15:434. doi: 10.1186/s12885-015-1461-0 (PMC4445803; doi:10.1186/s12885-015-1461-0)
Supplement: Additional file 7: Figure S2. — Correlation between individual genetic ancestry and global DNA methylation in leukocytes of cancer patients. (a) The African ancestry component was negatively correlated with DNA methylation (r = −0.187, p < 0.005). (b) The European ancestry component was positively correlated with DNA methylation (r = 0.169, p < 0.01). [file 12885_2015_1461_MOESM7_ESM.doc]

**ADDITIONAL FILE 7**


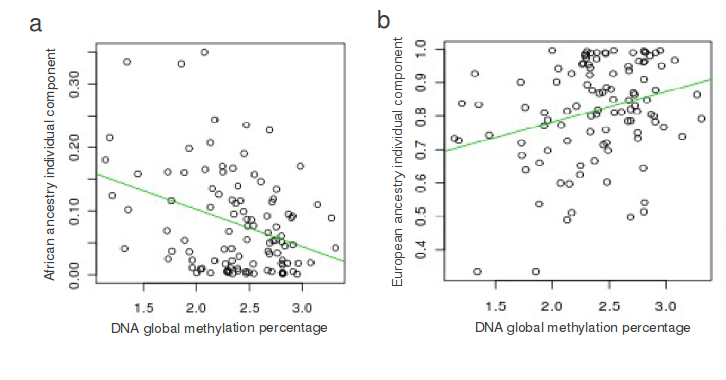
**Figure S2. Correlation between individual genetic ancestry and global DNA methylation in leukocytes of cancer patients.** (a) The African ancestry component was negatively correlated with DNA methylation (r = -0.187, p<0.005). (b) The European ancestry component was positively correlated with DNA methylation (r = 0.169, p<0.01).
