# Supplementary material for: Effect of genetic ancestry on leukocyte global DNA methylation in cancer patients
Source: BMC Cancer. 2015 May 27;15:434. doi: 10.1186/s12885-015-1461-0 (PMC4445803; doi:10.1186/s12885-015-1461-0)
Supplement: Additional file 8: Table S4. — Mean DNA methylation level (β) of CpG sites flanking AIMs in African Americans (AA), Caucasian Americans (CA) and Han Chinese Americans (HC). [file 12885_2015_1461_MOESM8_ESM.doc]

**ADDITIONAL FILE 8**

**Table S4.**  Mean DNA methylation level (β) of CpG sites flanking AIMs in African Americans (AA), Caucasian Americans (CA) and Han Chinese Americans (HC).

|  | **Mean β value** | | |  | **P value*** | |  |
| --- | --- | --- | --- | --- | --- | --- | --- |
| **AIMs** | **AA** | **CA** | **HC** | **AA_CA** | **AA_HC** | | **CA_HC** |
| rs1004704  rs10131076  rs1013459  rs10214949  rs10484578  rs10486576  rs10491097  rs10491654  rs10498255  rs10500505  rs10508349  rs10515535  rs10520678  rs12953952  rs1395771  rs1397618  rs1398829  rs1426654  rs1451928  rs1470524  rs1934393  rs1984473  rs2253624  rs257748  rs2585901  rs2817611  rs30125  rs3828121  rs4013967  rs4034627  rs4076700  rs4130513  rs4733652  rs4762106  rs6569792  rs6684063  rs6804094  rs6911727  rs798887  rs842634  rs868179  rs879780  rs888861  rs9310888  rs9320808  rs9323178  rs9325872  rs948360 | 0.5936  0.5763  0.3404  0.2534  0.3603  0.7203  0.6213  0.8356  0.5152  0.7393  0.8531  0.2757  0.4297  0.8638  0.6484  0.3733  0.6702  0.5420  0.7496  0.2731  0.3731  0.6653  0.4397  0.1631  0.8303  0.6034  0.2751  0.5444  0.0483  0.7093  0.5662  0.8339  0.6649  0.6630  0.3822  0.5230  0.5166  0.7234  0.5976  0.1213  0.2977  0.5897  0.3734  0.2495  0.3003  0.3587  0.4573  0.3999 | 0.5739  0.5259  0.3305  0.2375  0.3564  0.6588  0.5879  0.8319  0.5160  0.6816  0.8327  0.2571  0.3832  0.8656  0.6282  0.3771  0.5705  0.5301  0.7541  0.2569  0.3475  0.6252  0.3629  0.1166  0.8381  0.5604  0.2615  0.4294  0.0489  0.6458  0.5787  0.8256  0.6396  0.6052  0.3736  0.4849  0.4973  0.6851  0.5695  0.1223  0.2945  0.5740  0.3709  0.2352  0.2943  0.3550  0.3880  0.3981 | 0.6046  0.5601  0.3426  0.2615  0.3598  0.7301  0.6311  0.8455  0.5236  0.7390  0.8688  0.2737  0.4438  0.8586  0.6662  0.3775  0.6839  0.5428  0.7482  0.2690  0.3778  0.6761  0.4583  0.1364  0.8432  0.6228  0.2752  0.5671  0.0490  0.7384  0.5777  0.8367  0.6787  0.6760  0.3817  0.5236  0.5363  0.7490  0.6073  0.1216  0.3176  0.5871  0.3958  0.2546  0.2951  0.3588  0.4708  0.4060 | 0.0770  0.0003  0.9848  0.0166  0.9009  4.0681e-14  1.3212e-08  0.9848  0.7756  5.0857e-13  0.0166  0.6869  0.1716  0.7756  0.1644  0.5555  2.7353e-06  0.6129  0.6215  1.6714e-10  0.3428  0.0001  0.0059  2.8565e-08  0.5031  1.8760e-07  0.0313  3.1515e-05  0.8284  1.7973e-09  0.6215  0.6435  0.0604  9.0222e-06  0.9009  0.0414  0.3479  9.0222e-06  0.0001  0.7756  0.7756  0.0508  0.7756  0.6215  0.9851  0.9620  0.0011  0.7756 | 0.7078  0.6063  0.9269  0.0345  0.8104  0.9269  0.6063  0.8773  0.8104  0.8104  0.6063  0.8104  0.8104  0.6286  0.6121  0.9504  0.9269  0.9647  0.9269  0.5410  0.9269  0.6121  0.9269  0.8104  0.9269  0.1493  0.8104  0.5410  0.9269  0.6063  0.7228  0.9269  0.9269  0.9269  0.9269  0.9504  0.5410  0.5420  0.6063  0.9269  0.5410  0.7228  0.1882  0.9269  0.6286  0.8104  0.8104  0.6063 | | 0.0035  0.0080  0.8437  2.2900e-09  0.3899  2.7337e-16  3.5620e-13  0.6180  0.8437  2.8916e-12  8.4403e-05  0.8437  0.0188  0.1680  0.0062  0.3814  9.5370e-08  0.4353  0.3009  2.4789e-17  0.2156  2.9514e-07  0.0003  5.4496e-08  0.5092  2.5248e-15  0.0025  2.9123e-08  0.8480  1.8431e-15  0.8437  0.3930  0.0137  4.2145e-07  0.9461  0.0211  0.0045  7.5241e-11  1.1279e-07  0.4353  0.0547  0.3009  0.0619  0.3361  0.3219  0.5092  1.9236e-05  0.1217 |
|  |  |  |  |  |  |  | |

*Wilcoxon Rank Sum test, FDR p-value for the comparison of mean beta values between populations.
